# An Examination of Ironic Effects in Air-Pistol Shooting under Pressure

**DOI:** 10.3390/jfmk4020020

**Published:** 2019-04-08

**Authors:** Recep Gorgulu

**Affiliations:** Elite Performance in Sport Research Group, Faculty of Sports Sciences, Bursa Uludag University, 16059 Bursa, Turkey; gorgulurecep@gmail.com or

**Keywords:** ironic error, anxiety, pressure, instruction, motor control, pistol shooting, performance

## Abstract

This study aimed to test the incidence of ironic performance errors in elite air-pistol shooters. Previous research has revealed that, when shooters are anxious, avoidant instructions can cause ironic performance breakdown, especially in the unintended direction. Fifty-seven experienced air-pistol shooters were given specific instructions not to shoot to a certain part of a target, under low- and high-anxiety conditions, respectively. Results demonstrated that, when instructed not to shoot in a specific direction, anxious shooters did so a significant number of times. Interestingly, there was no difference in non-target non-ironic error, which provides specific support for Wegner’s theory of ironic processes of mental control in air-pistol shooting. Consequently, these findings illustrated that the combination of increased anxiety with avoidant instructions could lead to such unintended performance errors, called ironic error. Thus, understanding the mechanism of the anxiety-performance relationship may be a useful theoretical framework which could provide practical, instruction-based interventions to reduce susceptibility to ironic errors under pressure.

## 1. Introduction

The influence of anxiety on motor performance is one of the main areas of interest in performance psychology (e.g., [1,2,3,4]). Researchers have performed a great deal of research on determining the nature of the anxiety-performance relationship via conscious processing hypothesis [5,6], attentional control theory [7], and catastrophe models [8,9,10]. However, these theories do not offer a mechanism through which anxiety can precisely elicit *counter-intentional* errors. Wegner’s [11] theory of ironic processes of mental control predicts that, when under mental load, one’s thoughts will break down in a precisely counter-intentional manner. These errors are more severe than general errors and represent the worst possible scenario; that is, making a mistake that one least wants to make [12]. Such counter-intentional errors can be explained through Wegner’s [11,13,14,15] theory which, surprisingly, few researchers have examined whether Wegner’s theory that can be applied to explain counter-intentional errors in motor performance under pressure. When given an avoidant instruction, the incidence of counter-intentional movement does not only occur due to perceptual dysfunction or poor motor skills [11,16], but efforts to control these attempts intentionally, while insufficiently cognitively resourced (e.g., high mental load, anxiety, time pressure), can cause counter-intentional movement errors. Foundational to Wegner’s theory [11] is the premise that mental control requires two processes to work effectively. First, the cyclical operating process carries out intentional, effortful regulation by consciously searching for, and directing the individual toward, mental contents that will yield the desired outcome, or intended emotional state, known as the desired state. This operating process is easily interrupted and, thus, it is through active engagement in this mentally-demanding search, that regulation will most likely be maintained and the desired state will be reached. Second, the monitoring process subconsciously searches for mental contents that indicate a failure to achieve the desired state. If this monitor identifies any such failures, it reactivates the operating process with the aim of filling the mind with mental contents that are relevant to the desired state and, thus, re-establishing a regulated mind. Both processes work with a single control system and operate together as part of a feedback loop that, under normal circumstances, provides effective mental control [11].

Wegner [11] suggests that the very processes that enable an individual to regulate mental control are also, under certain conditions (e.g., anxiety, time pressure), inherent in undermining intentional mental control. Specifically, under pressure conditions, where cognitive resources are critically depleted (e.g., by a concurrent mental task, stress, or anxiety), the ability of the operator to focus attention solely toward wanted thoughts becomes limited, leaving the conscious mind hypersensitive to unwanted thoughts supplied by the monitor [16]. In other words, competing resources (e.g., anxiety) take some of the cognitive space that is required for the effortful operating process to operate effectively. As such, the operating process becomes less effective at introducing the desired content into awareness. Conversely, the functioning of the monitoring (unconscious and not easily interrupted) process remains largely unaffected under conditions of mental control. Thus, under mental load, the monitoring process becomes more salient and the search for thoughts or sensations that conflict with the desired state are sometimes enough to bring them into consciousness; thus, undermining the intended control [11]. This is precisely ironic because the monitoring process, that normally ensures that the to-be-avoided state is kept at bay, is the very process that increases the awareness and likelihood of the to–be-avoided state emerging [17,18].

Wegner and his colleagues have amassed an impressive body of evidence in support of this theory of ironic effects, most notably in the area of thought suppression [19,20]. Typical tests of the theory in this context involve asking participants to think of something, and not to think of something else, while subjecting them to either a high, or a low, mental load [11]. The results of these studies consistently reveal that people tend to think more of the to-be-avoided thought, when under mental load.

Wegner and colleagues [21] provided some promising initial research evidence for such performance irony. In two laboratory experiments, under either mental or physical load, participants suffered from more ironic errors. Further, Dugdale and Eklund [22] found that the experienced dancers were less stable on a wobble board when instructed not to wobble under mental load condition. Similar effects have also been reported in more recent research involving a golf-putting task. Researchers found that repressors (who deny their anxiety level) putt the ball significantly farther beyond the hole when specifically instructed not to do so, under stress [16,23], and that changes in visual attention might play an important role in the production of such undesired putting errors [24,25]. Recently, Woodman and colleagues [17] adopted a dart-throwing task and divided the board into a central target (i.e., bulls-eye) and four equally-sized quadrants that extended out from the bulls-eye to the edge of the board. Participants were instructed to aim for the bulls-eye, while being particularly careful not to hit one of the quadrants (e.g., top-right zone). Results revealed that performance deteriorated from the low- to high-anxiety conditions, and was characterised by an anxiety-induced increase in the number of darts landing in the specifically to-be-avoided zone. Taken together, these findings provide encouraging support for Wegner’s [11] ironic processes theory as an explanation for counter-intentional errors, including those that occur under anxiety, in motor performance.

From the perspective of applying the theory of ironic processes of mental control to a performance setting, the current paper aimed to replicate the findings from Woodman and colleagues [17] with an air-pistol shooting task. Specifically, in air-pistol shooting, the pre-shot period is a crucial time during which the shooter must prepare for the ensuing shot-release by aiming and holding the pistol towards the target. This requires the brain to process relevant visual information concerned with correct positioning of the pistol [26] when aiming for the target [21] and shows the importance of the mental control strategy (e.g., using avoiding instruction) used by the shooter. Therefore, it is hypothesized that performance would suffer in a specifically ironic fashion when shooters are anxious. In particular, ironic errors while anxious should increase, and performance should suffer, as a result. Conversely, and importantly—in terms of providing specific support for Wegner’s [11] theory of ironic processes—the non-ironic error should not change across anxiety conditions if the anxiety conditions are successfully manipulated in an ecologically valid manner.

## 2. Materials and Methods

### 2.1. Participants

Participants were approached before the training camp of the Great Britain (GB) International Shooting Union and were invited to participate in the study on a volunteer basis. Fifty-seven experienced (*M_experience (years)_* = 9.59, *SD* = 6.48) participants (33 men, 24 women; *M_age_* = 27.49, *SD* = 3.45; 52 right-handed, 5 left-handed) completed an air-pistol shooting task. Written, informed consent was obtained from all participants, individually. The GPower 3.1. [27] power calculation software indicated that, by adopting an alpha of 0.05 and a sample size of 57, the current study was powered at 0.80 to detect significant differences between conditions for effect sizes exceeding *f* = 0.20 (i.e., small-to-medium size effects), by repeated measures analysis of variance (ANOVA) [28]. While there is limited previous data upon which to base these calculations, Gorgulu’s [29] test of ironic effects (*n* = 32), adopting a similar design, revealed large, within-subject effects (η_p_^2^’s = 0.25). Accordingly, if similar effects were to emerge here, this study was more than adequately powered to detect them.

### 2.2. Measures

#### 2.2.1. Mental Effort

The perceived mental effort was measured using the Rating Scale for Mental Effort [30]. This retrospective, one-dimensional visual analogue scale ranges from 3 (*No Mental Effort at All*), through 115 (*Extreme Mental Effort*), to 150 (*No Anchor*). Participants were required to mark the scale at the point that best reflected the amount of mental effort invested in the task performance. This measure correlates with psychophysiological measures of mental effort, such as heart rate variability [31,32] and event-related potentials [33], which previously demonstrated acceptable reliability in the laboratory (*r* = 0.88) and real-life work settings (*r* = 0.78; [30]).

#### 2.2.2. Anxiety

Participants completed the Mental Readiness Form-3 [34]. The MRF-3 comprises three single-item factors that require participants to express their feelings regarding how they feel right now, by responding to three 11-point, Likert-type scales. From left to right, the scales are anchored at extremes with not worried and worried for cognitive anxiety; not tense and tense for somatic anxiety; and not confident and confident for self-confidence. Thus, high scores represent high cognitive anxiety, high somatic anxiety, and high self-confidence, respectively. The MRF-3 is commonly used in anxiety and motor performance research as a valid measure of emotional state (e.g., [23,35,36,37]). Krane [34] reported significant correlations between the MRF-3 and the CSAI-2 (Competitive State Anxiety Inventory-2) ranging from 0.58 to 63 [38]).

#### 2.2.3. Cardiac Activity

In addition to self-reported (MRF-3) measures of anxiety, and to increase experimental rigor, psychophysiological indices of anxiety, namely heart rate (HR) and heart rate variability (HRV; SDNN and r-MSSD), were measured using a Polar V800 heart rate monitor (HRM) with a Polar H7 chest strap at a sampling frequency of 1000 Hz. Recordings were subsequently stored in the HR monitor and then analyzed using the Polar Flow-Sync Software (Polar) to import into Kubios HRV version 2.2 software [39] for offline analyses. Specifically, HR was computed, along with the standard deviation of R-wave to R-wave intervals (SDNN) and the root mean square of successive R-R intervals (r-MSSD). These measures (especially increased heart rate and decreased SDNN and r-MSSD) have previously been associated with elevated pre-competitive anxiety [35,40,41].

#### 2.2.4. Performance

**Figure 1:**
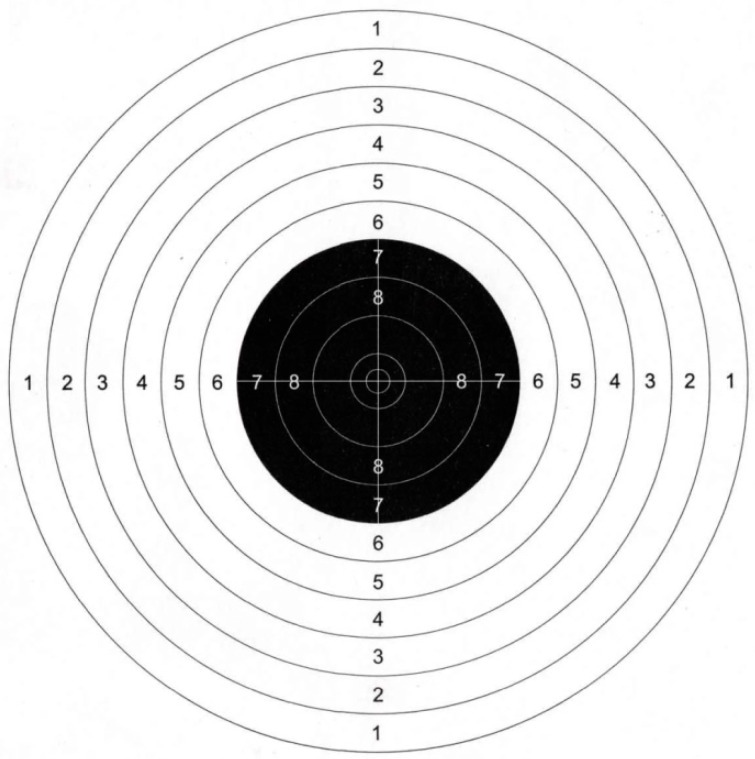
The 10-m air-pistol task.

Each participant was required to perform, individually and in the standing position, a 10 m pistol-shooting task, conforming to International Shooting Sport Federation (ISSF) rules and guidelines, with a 4.5 mm calibre air-pistol, at a competition target placed 10 m (11 yards) down range. Participants were allowed to use their competition equipment (pistol, clothing, etc.) over the course of the experiment. The standard, light-coloured cardboard target (17 × 17 cm; see Figure 1) with concentric score zones (the innermost zone worth 10 points, having a diameter of 11.5 mm) was used for easy scoring and collected by the experimenter after each trial. The target was positioned to the target line at 140 cm ± 5 cm from the floor and 10 m, horizontally, from the firing line.

### 2.3. Experimental Procedure

**Figure 2:**
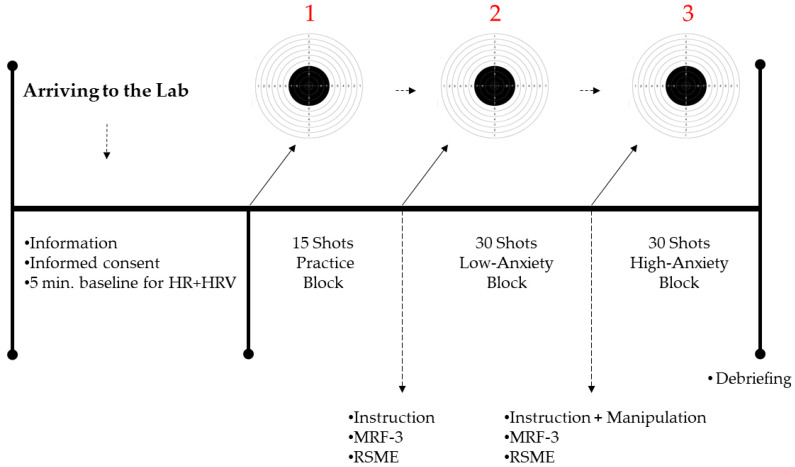
Demonstration of the experimental procedure.

Each participant individually attended a single laboratory session lasting approximately 60 min (see Figure 2). On arrival, using a standardized instructional set, the experimenter informed each participant of the procedure and described the scoring system for the pistol-shooting task. Participants then completed the consent form, with additional demographic data, before being fitted with the heart rate chest strap transmitter (Polar H7) connected with the HR monitor (Polar V800), which was worn by the experimenter.

Participants completed an air-pistol shooting task in three blocks (a. warm up; 15 shots, b. low anxiety condition; 30 shots, and c. high anxiety condition; 30 shots). The first warm-up block consisted of 15 practice shots. Scores were not recorded in this session. Following the warm-up, a 5-minute break was given and then participants received the first set of standardized instructions asking them to aim at a specific area of the shooting-target (a maximum score of 10) and to avoid another area. The order of presentation of the avoiding area (e.g., ironic error zone) was counterbalanced across the participants. Woodman and colleagues [17] measured the precision of ironic error in a dart-throwing task via two measures (e.g., radial error and arc-length of participants’ ironic error zone shots). The designated ironic error zone was one of the four quadrants (i.e., the top right of the target, excluding the area of the target zone that shot within the quadrant). More specifically, participants were told “please try to shoot the target (the innermost having a diameter of 11.5 mm, worth 10 points), or as close to the target as possible, but be careful not to shoot the top right quarter of the light-colored cardboard shooting target (17 × 17 cm), as you will score zero points each time you do so”. Immediately after the instructions were given, participants completed the MRF-3 and the self-reported mental effort (RSME) measure only once, then the instruction was repeated before the first shoot. The same procedure was used in the high-anxiety condition with one exception; before completing the MRF-3 and RSME, participants were informed that the highest scoring participant would receive £150 (approx. US$200). At the end of the experiment, participants were thanked and debriefed about the study.

### 2.4. Ethical Considerations

All procedures performed in this study involving human participants were carried out in accordance with the ethical standards of the University of Wales, ethics committee (process No: S/P05-14/15) and with the 1964 Helsinki declaration and its later amendments or comparable ethical standards.

## 3. Results

### 3.1. Experimental Anxiety Manipulation Check

Paired samples *t*-tests on the self-reported mental effort (e.g., RSME), anxiety (e.g., MRF-3), and psychophysiological reactions of anxiety (e.g., HR; HRV) confirmed the effectiveness of the anxiety manipulation. Specifically, the results demonstrated that participants’ cognitive anxiety (*t*56 = 5.98, *p* = 0.001) and somatic anxiety (*t*56 = 5.03, *p* = 0.001) were significantly higher, and their self-confidence (*t*56 = 2.85, *p* = 0.01) was significantly lower, in the high anxiety condition as compared to the low anxiety condition. Across anxiety conditions, results indicated that the participants’ mental effort did not change (*t*s < 1, *ps* > 0.5). Under the high-anxiety condition, participants’ heart rate was significantly higher (*t*53 = 2.86, *p* = 0.001) and the standard deviation of R-wave to R-wave intervals (SDNN; *t*53 = 5.70, *p* = 0.001) and root mean square of successive R-R intervals (r-MSSD; *t*53 = 6.50, *p* = 0.001) were significantly lower than the low-anxiety condition. The results are summarized in Table 1.

### 3.2. Performance

A 2 (anxiety: low, high) × 3 (zone: non-ironic error, ironic error, target) fully repeated measures ANOVA was conducted. There was no significant main effect for anxiety, *F*(1, 56) = 0.51, *p* = 0.27, ɳ2 = 0.07, a significant effect for error zone, *F*(2, 112) = 46.50, *p* < 0.001, ɳ2 = 0.42, and a significant anxiety x zone interaction, *F*(2, 112) = 11.34, *p* = 0.001, ɳ2 = 0.23, ε = 36. As the sphericity assumption was violated, the Greenhouse-Geisser correction factor to the degrees of freedom was also applied. Bonferroni-corrected follow-up paired sample *t*-tests revealed that, when anxious, participants fired fewer shots in the target (*t*56 = 6.45, *p* < 0.001) and more shots in the ironic error zone (*t*56 = 5.92, *p* < 0.001), as hypothesized. Importantly, there was no significant non-target non-error difference between the high- and low-anxiety conditions, regardless of which of the three remaining quadrants was conceptualized as a non-ironic error (*t*s < 1, *p*s > 0.5).

### 3.3. The Precision of Ironic Error

A repeated measure of Multivariate Analysis of Variance (MANOVA) was conducted specifically on the ironic error data under low- and high-anxiety conditions. First, each participant’s mean radial error was taken within the ironic zone as the measure of the distance from the target zone. Second, each participant’s mean arc length was taken within the ironic zone (from the closest non-ironic zone) as the measure of the distance of the ironic error zone. Contrary to previous research, the multivariate difference between low- and high-anxiety conditions was not significant; Wilks’s L = 0.56, *F* (2, 42) = 0.76, *p* = 0.54.

## 4. Discussion

The aim of the present research was to test Wegner’s [11] ironic processes of mental control theory and to replicate the findings from previous research [17] in an air-pistol shooting task. The results of the present research show that the performers suffered significant ironic errors of performance when they were anxious, which is in line with previous research [17,22,23,42,43]. Especially when negatively primed (e.g., do not hit the top right quarter of the target in pistol shooting), anxious shooters do not suffer error of a diffuse nature, but rather, suffer specifically to counter-intentional error. The results of the present research provide support for Wegner’s theory that anxious individuals make more ironic errors compared to when they are not anxious. However, the latter aim was to understand the nature of ironic performance errors by replicating the methodology of Woodman and colleagues [17] and examining how precise the ironic errors are, under different anxiety conditions (e.g., low and high). The findings of the present research did not demonstrate a difference in terms of specific ironic errors.

To date, Wegner’s theory of ironic processes of mental control has been measured and observed over numerous trials in sport, more notably in laboratory-based studies (e.g., [17,21,44]) and a few field-based studies (e.g., [35,42]). This research, however, demonstrates that ironic performance errors can be seen in experienced air-pistol shooters, rather than novices, suggesting that this is a meaningful and robust potential concern for those who are required to perform under such pressurized situations (e.g., competition). Although the present research supports Wegner’s [17] theory, there are other studies that provide some contradictory results to the ironic processes of mental control theory. First, researchers found that under high cognitive load conditions, when golfers were instructed negatively with “not to putt short of the hole” they overcompensated by putting the golf ball significantly farther than under conditions of lower cognitive load [45]. Recently, Malhotra and colleagues [46] found similar results in a driving study (e.g., tourist drivers on the unfamiliar side of the road). Participants were instructed “not to drift towards the centre line” while subjected with high cognitive load (e.g., tone counting). The results did not demonstrate ironic behavior under high cognitive load. In fact, the results of the study were associated with over-compensatory behavior, with participants driving further from the center line compared to those who received neutral instruction [46]. However, tone counting while driving on the unfamiliar side of the road is not an ecologically valid method therefore, this method may have failed to tax the cognitive resources of the participants [17]. Thus, future research should be undertaken to ensure that participants’ cognitive resources are significantly taxed in an ecologically valid manner [17] in order to manipulate a certain situation while observing ironic behavior.

The main limitation of the present research into the occurrence of ironic performance errors is the lack of consideration given to whether ironic errors occur due to negative instructions or simply, uniformly, due to anxiety. For example, the results obtained from the mental effort measure (RSME) was not different from low to high-anxiety condition, which shows that mental effort might not be a clear indication of anxiety in the present study. However, the results of the present study also showed that there was an increase in the heart rate variability under high-anxiety conditions, as compared to the low-anxiety conditions. The heart rate variability has been used in previous research as a successful indicator of a physiological response to anxiety (e.g., [41,47,48]). Therefore, an increase in the incidence of ironic performance errors would be related to the elevated anxiety rather than the solely negative instruction. According to Wegner’s theory (e.g., ironic processes of mental control) in certain condition some of the cognitive space is required for the operating process to operate effectively and allow the individuals to achieve the desired state would be taken up by competing resources (e.g., anxiety). Under mental load, when participants were instructed with negative words (e.g., do not shoot the top right quarter of the target), the monitoring process may become more salient and start searching for conflictive thoughts with the desired state, which may sometimes be enough to bring them into consciousness and, therefore, undermine the intended control [17]. Conjointly, using negative instruction in order to pay attention to not do a specific action may, ironically, increase performers’ awareness and the likelihood of the undesired action under mental load [29]. Consequently, one may be more susceptible to doing what one intends to avoid, when one least wants to do it, counter-intentionally. Therefore, taking a deep breath and trying not to hurry, when under pressure, could allow performers an extended duration of programming while minimizing distractive or unwanted thoughts [49].

## 5. Conclusions

In conclusion, the combination of a high-pressure situation with negative instructions is potentially fraught with undesirable consequences that are perversely predictable. The present research has examined whether consciously attempting to avoid certain behaviors would lead to counter-intentional error in a target-based motor control study especially when anxious (e.g., pistol-shooting). The results from the current study may lead to the creation of more efficient coaching techniques and effective coaching plans for those performers who may work with instructive technical skills (e.g., shooting, dart-throwing). In summary, the present research demonstrates that ironic performance errors can be observed in different performance contexts such as reaction time [50] or externally paced tasks, indicating that it is a meaningful and robust potential concern for performers who are required to perform under pressure (e.g., Olympic athletes, police officers, surgeons, etc.). Considering this, coaches, practitioners, and sport psychologists would do well to be particularly careful with the specific instructions they use, as part of their coaching, when helping performers to ensure that they do not contribute to the likelihood of mental control backfiring, when it matters most to the performer [17]. Thus, future research should continue to investigate the mechanism of the anxiety-performance relationship, which may be a useful theoretical framework that could provide practical, instruction-based interventions to reduce susceptibility to ironic errors under pressure.

## Figures and Tables

**Table 1:** Descriptive statistics confirming the effectiveness of the anxiety manipulation.

	Condition		
Measure	Low-Anxiety	High-Anxiety	
	Mean (SD)	Mean (SD)	*t*(56)
Cognitive anxiety	6.56 (2.48)	8.24 (2.25)	5.98 ***
Somatic anxiety	6.18 (2.27)	7.83 (2.64)	5.03 **
Self-confidence	6.23 (1.93)	5.20 (1.97)	2.85 **
Effort	82.65 (19.40)	98.43 (20.35)	9.54
			*t*(53)
Heart rate (bpm)	81.75 (18.15)	94.58 (16.40)	2.86 *
SDNN (ms)	65.13 (21.75)	53.47 (22.63)	5.70 **
r-MSSD (ms)	46.38 (25.41)	31.33 (18.47)	6.50 **

Notes: * *p* < 0.05, ** *p* < 0.01, *** *p* < 0.001.
